# microRNA-144 functions as a diagnostic and prognostic marker for retinoblastoma

**DOI:** 10.6061/clinics/2020/e1804

**Published:** 2020-08-14

**Authors:** Qian Zheng, Qin Zhu, Cuiping Li, Shuang Hao, Jianguo Li, Xin Yu, Dengmei Qi, Yu Pan

**Affiliations:** Department of Ophthalmology, Zibo Maternal and Child Health Hospital, Zibo 255029, China.

**Keywords:** Retinoblastoma, miR-144, Diagnosis, Prognosis

## Abstract

**OBJECTIVES:**

Retinoblastoma (RB) is a highly malignant eye tumor with a low survival rate and a high metastatic rate. The current work was designed to investigate the potential roles of microRNA-144 (miR-144) in the diagnosis and prognosis of RB.

**METHODS:**

miR-144 expression levels in RB tissues and adjacent normal tissues, as well as serum samples from RB patients and healthy controls were measured. The association between miR-144 expression levels and clinical features were analyzed. Moreover, diagnostic and prognostic values of miR-144 in RB were verified by receiver operating characteristic analysis and Kaplan-Meier survival assays.

**RESULTS:**

The expression level of miR-144 was markedly decreased in tumor tissues of RB patients, and the expression level of miR-144 was positively associated with tumor size and metastasis in RB patients. Moreover, miR-144 can distinguish tumor tissues from normal tissues with high specificity and sensitivity, and RB patients with lower miR-144 expression have shorter overall and disease-free survival rates than those with higher miR-144 expression. Alternatively, miR-144 also decreased in the serum of RB patients in comparison with healthy subjects, and miR-144 expression levels in the tissue samples and serum were positively correlated. Furthermore, miR-144 levels in the serum of RB patients sensitively distinguished RB patients from healthy controls.

**CONCLUSIONS:**

miR-144 expression was downregulated in serum and tissue samples of RB patients and may function as a diagnostic and prognostic marker for RB.

## INTRODUCTION

Retinoblastoma (RB) is a common eye malignant tumor and the most common type of tumor in children. According to previous studies, RB shows rapid growth, a high metastatic rate, and poor prognosis (1-3). With the development of modern technology, the survival rate of RB has increased to over 50% (4,5). However, long-term prognosis of RB patients is still unfavorable, and among children who survive, some lose their vision (1). Therefore, it is important to explore the underlying mechanisms involved in RB tumorigenesis, development, and prognosis.

MicroRNAs represent a class of short non-coding RNAs with a length of approximately 20 nucleotides. MicroRNAs (miRNAs) are able to bind to the 3′ UTR of their target genes and inhibit the expression of the target genes at the posttranscriptional level (6,7). Accumulating evidence has reported that miRNAs regulate different cellular behaviors, e.g., cell growth, apoptosis, migration, invasion, and differentiation (7-9). According to various studies, several miRNAs (miR-936, miR-23-5p, miR-140-5p) were dysregulated in RB patients, thereby mediating the development and metastasis of RB (10-14).

MicroRNA-144 (miR-144) is a member of the miRNA family. Dysregulation of miR-144 was verified in various tumors; miRNAs function either as tumor suppressors or onco-miRNAs (15-19). However, the role of miR-144 with regard to the diagnostic and prognostic values of RB has not yet been elucidated. Hence, the aim of this study was to explore the potential clinical value of miR-144 for the diagnosis and treatment of RB.

## MATERIALS AND METHODS

### Samples

A total of 50 RB tumor tissues and paired adjacent tissues were used in this study. Samples were obtained from children hospitalized at Zibo Maternal and Child Health Hospital who underwent enucleation surgery between July 2011 and May 2014. In addition, serum samples of these patients and 50 healthy controls were also collected. Written informed consent was provided by all study participants. This study was approved by the Ethics Committee of Zibo Maternal and Child Health Hospital. All RB patients were confirmed based on clinical manifestations and imaging results without receiving any adjuvant therapy prior to surgery. Samples were collected from all participants and frozen at -80°C for subsequent examination.

Clinical information of patients was collected and is shown in Table 1. The 5-year follow-up analysis was updated telephonically every 5 months.

### Real-time PCR

To examine the expression level of miR-144, real-time PCR was performed. Briefly, total RNA was isolated from tissue and blood samples using the TRIzol reagent (Invitrogen, Carlsbad, USA). Concentrations of RNA samples were evaluated based on their absorbance ratio at 260 nm/280 nm, according to the manufacturer’s instructions (ThermoScientific NanoDrop Technologies, Wilmington, DE, USA). Thereafter, cDNA was reverse-transcribed using the commercially available Reverse Transcription Kit (Invitrogen). The PCR was conducted using the ABI 7500 system (Applied Biosystems, Inc., USA) with the SYBR Green kit (Invitrogen) according to the manufacturer’s instructions. miR-144 expression in each sample was normalized to that of U6. The primer sequences used were: miR-144 forward, 5′-TGCGGTACAGTATAGATGAT-3′ and reverse, 5′-CCAGTGCAGGGTCCGAGGT-3′; U6 forward, 5′-TGCGGGTGCTCGCTTCGGCAGC-3, and reverse 5′-CCAGTGCAGGGTCCGAGGT-3′.

### Statistical analysis

SPSS 22.0 and GraphPad statistical software were used for data analysis. All experimental data were expressed as the means ± standard deviations. The differences between two groups were compared using the Student’s t-test. The diagnostic value of miR-144 was evaluated by receiver operating characteristic (ROC) curve analysis. The Kaplan-Meier was used to determine the overall survival (OS) and disease-free survival (DFS) of patients. The clinical information in Table 1 was analyzed by the chi-square test. *p-*values less than 0.05 were considered statistically significant.

## RESULTS

### Downregulation of miR-144 expression in RB tumor tissues compared with non-tumor tissues

**Figure 1:**
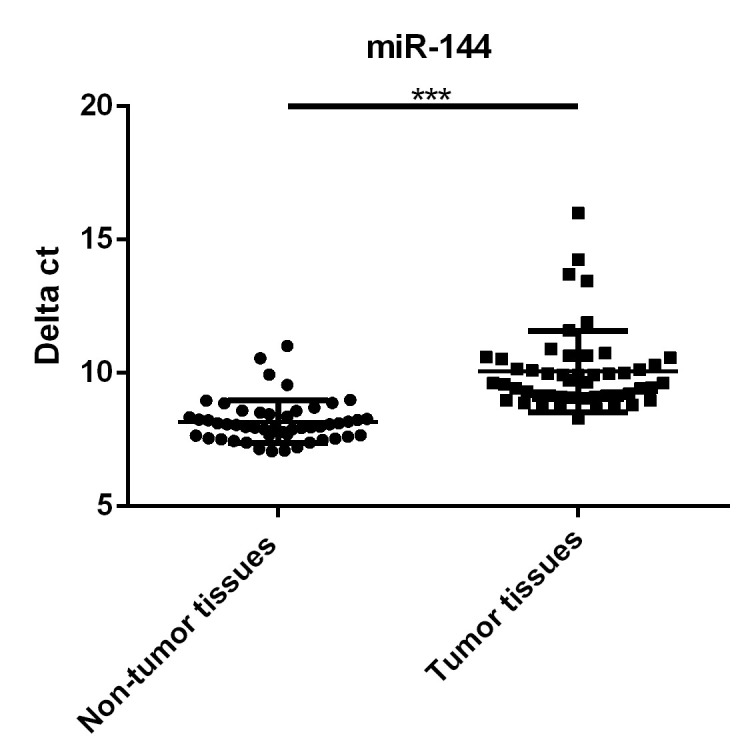
Downregulation of miR-144 in retinoblastoma (RB) tumor tissues, in comparison with non-tumor tissues. ***p<0.001.

miR-144 levels in the tumor samples of 50 RB patients and 50 matched non-tumor adjacent tissue samples were compared by RT-qPCR assays. As shown in Figure 1, the miR-144 level markedly decreased in RB tumors in comparison with non-tumor tissue (*p*<0.001). Moreover, RB patients have been divided into the miR-144-high group (n=23, delta ct of miR-144≤median value 9.6) and miR-144-low group (n=27, delta ct of miR-144>median value 9.6) based on miR-144 levels. As seen in Table 1, we found that decreased miR-144 levels may indicate increased tumor size (*p*<0.05), advanced clinical stage (*p*<0.01), and an increased chance of metastasis (*p*<0.01).

### MiR-144 expression in RB tumor tissues may serve as a diagnostic and prognostic marker for RB

**Figure 2:**
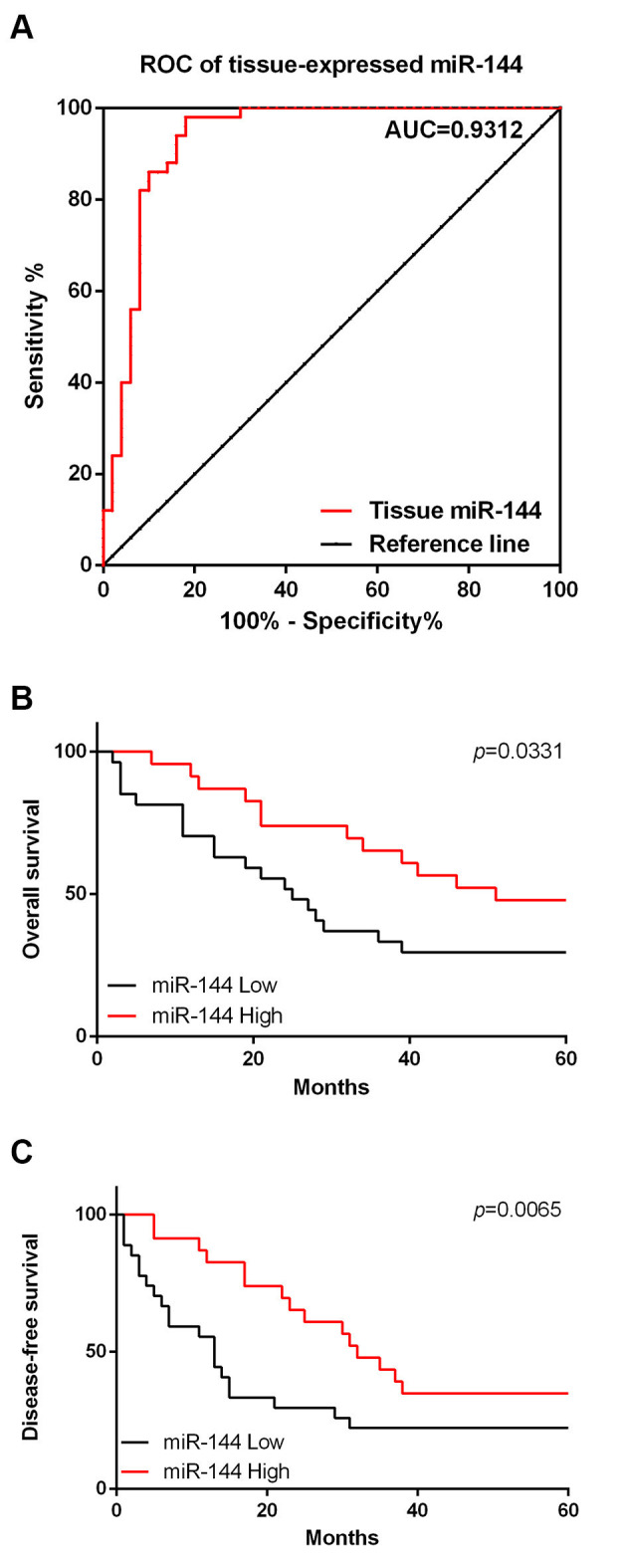
miR-144 expression in retinoblastoma (RB) tumor tissues may serve as a diagnostic and prognostic marker for RB. A. Results of receiver operating characteristic (ROC) analysis. B. Overall survival of RB patients. C. Disease-free survival of RB patients.

ROC analysis was performed to analyze the potential diagnostic value of miR-144 for RB. As shown in Figure 2A, the area under the curve (AUC) of miR-144 was 0.9312 (95% confidence interval 0.8765 to 0.9859; cut-off value, 8.634; sensitivity, 98%; specificity, 82%), suggesting that the miR-144 level is a sensitive biomarker for the diagnosis of RB; moreover, the prognostic value of miR-144 was also analyzed by the Kaplan-Meier method. We found that during the 5-year follow-up period, the miR-144 low group decreased the OS (Figure 2B, *p*=0.0065) and DFS, when compared with the miR-144-high group (Figure 2C, *p*=0.0331).

### Decreased miR-144 expression in the serum of RB patients

**Figure 3:**
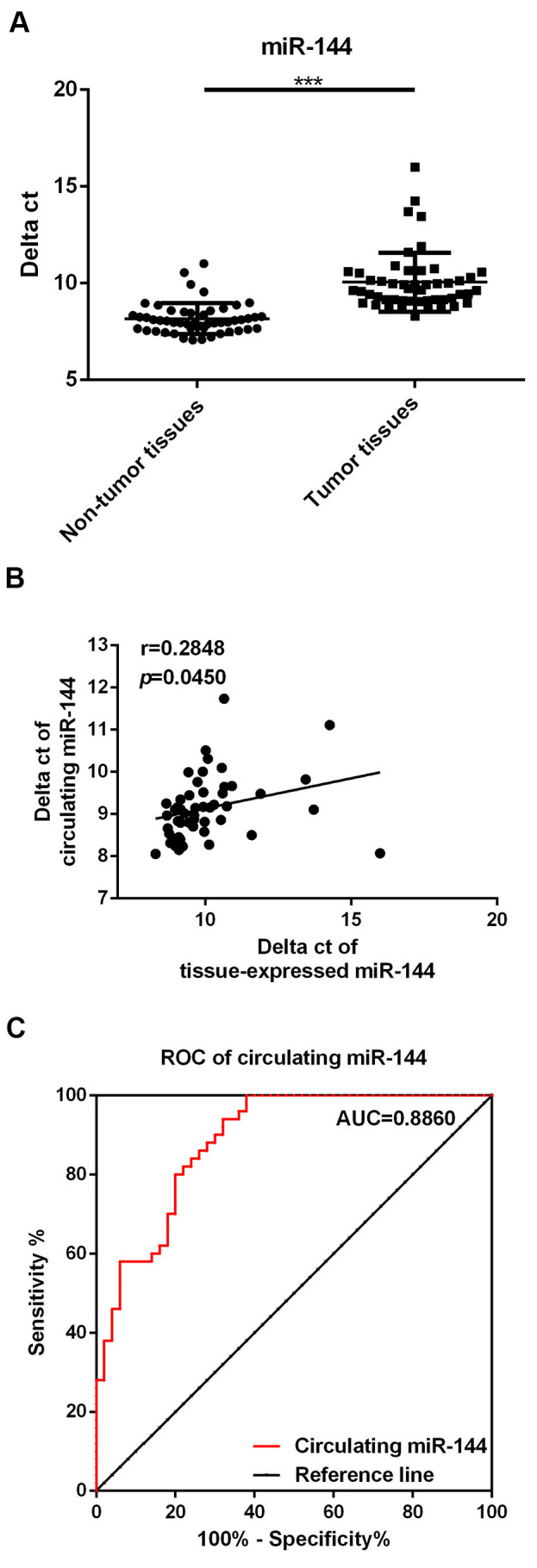
Decreased miR-144 expression in the serum of retinoblastoma (RB) patients. A. Comparison of miR-144 levels in serum of RB patients and healthy controls. B. Correlation between miR-144 levels in RB tumor and serum samples of RB patients. C. Results of receiver operating characteristic (ROC) analysis for circulating RB. **p<0.01.

miR-144 levels in serum samples of 50 patients and healthy controls were also compared. It was observed that miR-144 levels decreased markedly in serum samples of RB patients in comparison with the healthy controls (Figure 3A, *p*<0.01). Moreover, correlation analysis demonstrated that miR-144 levels in tissue and serum samples of RB patients were positively correlated (Figure 3B, r=0.2848, *p*=0.0459).

### Circulating miR-144 levels may serve as a potential diagnostic marker for RB

Finally, we performed ROC analysis to determine the potential diagnostic value of circulating miR-144 levels in distinguishing RB patients from healthy controls. As shown in Figure 3C, the AUC of circulating miR-144 was 0.8860 (95% confidence interval, 0.8232 to 0.9488; cut-off value, 8.499; sensitivity, 80%; specificity, 80%), suggesting that circulating miR-144 is a sensitive biomarker for the diagnosis of RB.

## DISCUSSION

The current work focused on the roles of miR-144 in the pathogenesis of RB. We observed that miR-144 expression was markedly decreased in tumor tissues and serum samples of RB patients, and that miR-144 may function as a potential diagnostic and prognostic biomarker.

Numerous studies have suggested that dysregulation of miR-144 may contribute to the development of different types of cancers. For instance, it has been reported that miR-144 could inhibit growth and metastasis of breast cancer cells by targeting CEP55 (15). Moreover, miRNA-144 has been reported to regulate the carcinogenic behavior of gastric cancer cells (19) and alleviate the cisplatin resistance of cervical cancer cells (20). However, whether miR-144 is involved in the pathogenesis of RB remains unclear. In the present study, we found that miR-144 expression was markedly decreased in RB tumor tissues. This decreased level of miR-144 was associated with increased tumor size, advanced clinical stage, as well as increased metastasis. Overall, our data suggested that miR-144 expression was downregulated in RB and that it may function as a tumor suppressor.

Increasing evidence has proposed the potential use of miRNAs as diagnostic and prognostic biomarkers for cancers. The potential diagnostic and prognostic value of miRNAs, and the roles of miRNAs in RB as biomarkers have been discussed previously (21). Alternatively, the roles of miR-144 as potential biomarkers in other cancers have also been discussed. In the present study, we found that the AUC of miR-144 was 0.9312 suggesting that miR-144 is a sensitive biomarker for distinguishing RB tumor tissues from adjacent normal tissues. Moreover, results of the survival analysis indicated that decreased miR-144 expression may indicate poor prognosis. Therefore, the results of the current study suggested that miR-144 may function as a potential diagnostic and prognostic biomarker for the diagnosis and treatment of RB.

The above data has indicated the diagnostic value of miR-144 levels in RB tumors; however, in clinical situations, it is inconvenient to obtain tissue samples for diagnostic purposes. A few recent studies have suggested that miRNAs can be released by tumor tissue and that their levels can be stably maintained in the blood. Therefore, to detect miRNA expression in blood samples (so-called circulating miRNAs) may be a cheap and easy method for the early diagnosis of different diseases (22-24). Studies on circulating miRNAs with regard to RB are limited. Zhou et al. suggested that miR-338-5p levels in the serum of RB patients may function as potential biomarkers (21). Notably, we found that circulating miR-144 levels were also downregulated in RB patients, and that the expression of miR-144 in RB tumor tissues and that in serum samples were negatively correlated. These results suggested that the aberrant decrease in miR-144 expression in the serum of RB patients was primarily due to the formation of tumor tissues. Moreover, the results of ROC analysis confirmed the diagnostic value of circulating miR-144 levels for RB, which was consistent with levels of tissue-expressed miR-144.

In summary, the present work revealed that decreased miR-144 expression may serve as a potential diagnostic and prognostic biomarker for RB. However, these results require confirmation using a larger sample size in future investigations.

## AUTHOR CONTRIBUTIONS

Zheng Q was responsible for the data curation, investigation, methodology, and writing manuscript original draft. Zhu Q was responsible for the formal analysis, investigation, methodology, and validation. Li C was responsible for the formal analysis, methodology, and software resources. Hao S was responsible for the data curation and software resources. Li J was responsible for the investigation, methodology, and validation. Yu X was responsible for the data curation, methodology, and software resources. Qi D was responsible for the formal analysis, and investigation. Pan Y was responsible for the conceptualization, manuscript original draft, editing and review.

## Figures and Tables

**Table 1:** Clinical information of retinoblastoma (RB) patients.

	Patients (n=50)	miR-144-high group (n=23)	miR-144-low group (n=27)	*p*-value
Age (years)				0.5196
≥5	39	17	22	
<5	11	6	5	
Gender				0.9819
Male	26	12	14	
Female	24	11	13	
Tumor size				0.0126*
<10 mm	21	14	7	
≥10 mm	29	9	20	
TNM stage				0.0034**
I-II	28	18	10	
III-IV	22	5	17	
Metastasis				0.0054**
Negative	20	14	6	
Positive	30	9	21	

*p<0.05

**p<0.01.
